# TLR2 polymorphisms, Arg753Gln and Arg677Trp, are not associated with increased burden of tuberculosis in Indian patients

**DOI:** 10.1186/1756-0500-2-162

**Published:** 2009-08-18

**Authors:** Debasis Biswas, Shailendra K Gupta, Girish Sindhwani, Abhishek Patras

**Affiliations:** 1 Department of Microbiology, Himalayan Institute of Medical Sciences, Swami Ram Nagar, Dehradun 248140, India; 2 Department of Bioinformatics, Indian Institute of Toxicology Research, PO Box 80, MG Marg, Lucknow 226001, India; 3 Department of Pulmonary Medicine, Himalayan Institute of Medical Sciences, Swami Ram Nagar, Dehradun 248140, India

## Abstract

**Background:**

In view of the role of TLR2 activation in host defense against mycobacteria, the present study was conducted to examine whether TLR2 polymorphisms could account for the increased prevalence of tuberculosis in Indian patients. Detection of such polymorphisms would help in assessing the risk of developing active tuberculosis among contacts or HIV positive patients and in identifying candidates for chemoprophylaxis.

**Findings:**

One hundred patients with tuberculosis and 100 controls were investigated for the presence of two TLR2 polymorphisms, viz. Arg753Gln and Arg677Trp, using PCR-RFLP of a 340 bp region of the TLR2 gene, followed by DNA sequencing of a randomly selected group of 35 patients. While these polymorphisms were found to be non-existent in our study groups, we observed a novel polymorphism Phe749Tyr in 2 patients. However, this polymorphism was associated with negligible deviation in Delphi electrostatic potential and structural alignment from the wild-type TLR2 protein, making it an unlikely candidate for any significant structural or functional alteration at the protein level.

**Conclusion:**

Hence we conclude that, contrary to reported associations in other populations, TLR2 polymorphisms are not responsible for the increased prevalence of TB in the Indian population.

## Research hypothesis

Of the different members of the Toll-like receptor (TLR) family, TLR2 has been shown to be a principal mediator of macrophage activation in response to mycobacteria. Defective TLR2 genes have been associated with suppressed macrophage response to mycobacteria in both animal and human studies. Expression of mutant TLR2 (Pro681His) in RAW murine macrophages inhibited TNFα production in response to both virulent and avirulent mycobacteria [1]. Comparing the resistance to airborne infection with M. tuberculosis in TLR2, TLR4, CD14 knockout and control mice groups, Reiling et al found that TLR2 knockout mice showed decreased resistance to TB on high-dose exposure [2]. In addition, two single nucleotide polymorphisms (SNPs) (Arg677Trp and Arg753Gln) of the TLR2 gene have been identified by Bochud et al that impaired the macrophage response to M. leprae and M. tuberculosis in humans [3]. While the Arg677Trp polymorphism was found to associate with TB in a Tunisian population [4] and with lepromatous leprosy in a Korean population [5], it was not detected among Caucasians [6]. The other SNP, Arg753Gln, was shown to significantly correlate with tuberculosis in a Turkish population; thereby signifying that polymorphism in the TLR2 gene demonstrated an ethnic variation [7].

Considering the fact that India is home to a third of the global cases of tuberculosis, we hypothesized that these SNPs in the TLR2 gene could contribute to the high burden of TB among Indian patients. Detection of such polymorphisms in tubercular patients would help in assessing the risk of developing active TB among contacts or HIV positive patients and in identifying candidates for chemoprophylaxis.

## Methods

One hundred patients suffering from Pulmonary TB were recruited from the Community Health Clinics and OPDs of Himalayan Institute of Medical Sciences, Dehradun, India. The diagnosis of Pulmonary TB in these patients was confirmed by sputum-smear microscopy done on three consecutive sputum samples. The control group included 60 healthy household contacts of the recruited cases and 40 healthy individuals without any past history of TB. The study design was approved by the institutional ethics committee, in concordance with national guidelines. After obtaining written informed consent, blood samples were collected from the patients and controls in EDTA vials and their clinical details were recorded. Genomic DNA was extracted from the blood samples, using QIAamp DNA Blood Minikit (Qiagen).

To detect the TLR2 polymorphism, a 340 bp region of the TLR2 gene was amplified, as previously reported [6] using the following primers: forward 5'-GCCTACTGGGTGGAGAACCT-3' & reverse 5'-GGCCACTCCAGGTAGGTCTT-3'. Approximately 40 ng sample DNA was added to a reaction volume of 25 μl containing 2.5 μl 10× buffer, 2 mM MgCl_2_, 0.2 μl deoxyribonucleoside triphosphate mix and 25 pmol of each primer. PCR was performed under the following conditions: 95°C for 10 mins, followed by 30 cycles of 95°C for 30 secs, 58°C for 30 secs, and 72°C for 25 secs, and a final elongation step of 72°C for 5 mins. Following PCR, 3 μl of the amplicon was incubated for 2 hours with 0.5 U *Aci*I in a total volume of 10 μl at 36°C. Samples were subjected to agarose gel electrophoresis to identify digestion patterns characteristic of wild-type and mutant TLR2.

To confirm the results of PCR-RFLP, DNA sequencing of the amplicons was performed on a randomly selected group of 35 patients at the Deptt. of Biochemistry, University of Delhi, India.

To sense the SNPs in the selected DNA sequences, multiple sequence alignment was performed using built in interface for ClustalW [8] in Genchek software version 2.4.1.5 from Ocimum Biosolutions. The gap opening, gap extension penalty were set to 15 and 6.66 respectively. DNA Identity scoring matrix was used for the alignment. To validate the result at protein level, all aligned sequences along with the gaps were translated to protein. Protein sequence of TLR2 gene (Accession no. NP_003255.2) was downloaded from the NCBI server [9]. TIR domain of TLR2 (Amino acid 639: 784) was analyzed for its structural detail. Homology modeling approach was followed to design the 3D structure of TIR domain using 1FYX|A and 1FYV|A with 'Build homology model' protocol based on modeler interface [10] available on Discovery studio. Mutant model was developed for the modeled structure of TIR domain by using 'Build mutants' protocol for the observed mutation (F749Y) in few samples. Both structures were analyzed for changes in electrostatic potential using Delphi [11] on Discovery studio. The program calculates the electrostatic properties for molecular system using a finite difference solution to the nonlinear Poisson-Boltzmann equation [12]. In Delphi analysis, one quantity that is often important is the change in pK at some location due to a site-directed mutation at another location. Change in pK due to change in amino acid group was calculated as eq (1).

(1)

φ_0 _= the potential at site *i *due to the original group

φ_m _= the potential at site *i *due to the mutated group

γ_i _= -1 or 1 for an acidic or basic group, respectively.

To solve the problem of change in pK, two DelPhi calculations were carried out, one for the original molecule to find φ_0_, and another to find φ_m_. In both calculations, the only charges present in the molecule are those on the group involved in the mutation. Since all of the other charges in the molecule are the same in both cases, the contribution to the potential at the site of interest due to these charges is also the same and, therefore, does not have to be included. Further, 3D structure of TIR and mutated model were superimposed to analyze any structural difference because of polymorphism found in some samples.

## Results

One hundred patients with TB and 100 controls (including 60 healthy household contacts of TB patients and 40 unrelated healthy volunteers) were investigated for the presence of the two TLR2 polymorphisms. The demographic and clinical characteristics of the study groups are summarized in Table 1.

**Table 1:** Demographic and clinical characteristics of the recruited subjects

	**Patients**	**Controls**
No. of subjects	100	100
Age (yrs): (Mean ± SD)	42.52 ± 20.94	35.78 ± 16.56
Male/Female	64/36	72/28
Past h/o TB	7	0
Family h/o TB (other than index case)	6	3
Duration of illness (months): (Mean ± SD)	19.2 ± 3.5	
Pulmonary TB	80	
< 3 lobes involved (%)	52 (65)	
≥ 3 lobes involved (%)	28 (35)	
Extra-pulmonary TB	20	

Screening for the presence of the two polymorphisms was done using PCR amplification of a 340 bp region of the TLR2 gene, followed by RFLP with *Aci*I enzyme. While wild-type TLR2, on restriction digestion, yielded a fragment of 227 bp, Arg753Gln and Arg677Trp polymorphisms were associated with fragments measuring 265 bp and 302 bp respectively. Among the patients, we did not find any of the polymorphisms in heterozygote or homozygote form. Similarly, none of the polymorphisms could be detected among the household contacts or the healthy controls (Table 2).

**Table 2:** Genotype distribution of TLR2 polymorphisms in recruited subjects

**Genotype**	**Patients** **(n = 100)**	**Controls** **(n = 100)**
TLR2 Arg753Gln		
-/-	100	100
+/-	-	-
+/+	-	-
TLR2 Arg677Trp		
-/-	100	100
+/-	-	-
+/+	-	-

**Figure 1:**
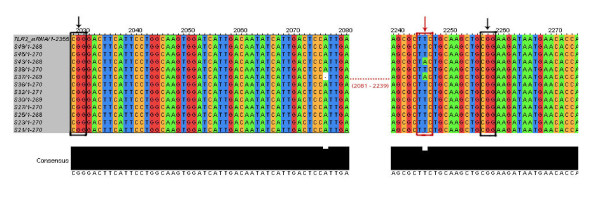
**Highly conserved multiple aligned sequences of a representative group of 12 patients along with TLR2 mRNA (NCBI accession no. NM_003264.3)**. Region of interest, for SNP detection were enclosed in Black rectangle showing absence of desired SNPs (G/A, C/T at position 2030 & 2257 respectively) in our samples. Further new SNP (T/A) detected in 2 samples at position 2246 (marked with red arrow).

To confirm the absence of the two polymorphisms, Arg677Trp and Arg753Gln in our patients, we undertook DNA sequencing of the PCR amplicons derived from 35 randomly selected patients, covering the region 2029 to 2258. We performed multiple alignment of PCR amplicons with TLR2 mRNA (NCBI accession no. NM_003264.3). We found the DNA sequences to be highly conserved among the patients (Figure 1).

**Figure 2:**
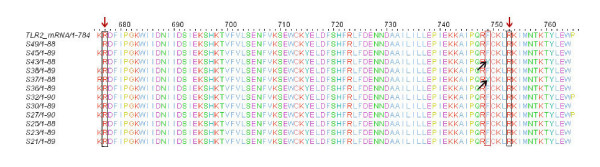
**Translated multiple alignment sequences along with gaps**. Positions 677 and 753 marked with red arrow showing highly conserved ARG. In two samples (S43, S37) amino acid PHE at position 749 mutated to TYR (Black arrow)

Entire multiple aligned sequences translated along with the gaps to analyze the result at protein level. In our patients, we found that position nos. 677 and 753 (figure 2) are conserved, confirming the absence of two polymorphisms (Arg677Trp, Arg753Gln). However, we found one polymorphism Phe749Tyr in two of the randomly selected samples (S37, S43), which was further analyzed with the help of bioinformatics tools.

3D structure of TIR domain designed using homology modeling approach by using engineered mutated toll like receptor 2 chain A (1FYX) and toll like receptor 1 chain A (1FYV) of *Homo sapiens *available with protein data bank [13]. Electrostatic potential of the model was evaluated using Delphi electrostatic calculation. Charges on individual atom in the model for Delphi calculation were taken from Charmm22 force field charge and atomic radii were taken from the Bondi radii set [14]. Same calculation was repeated for the mutated model (Phe749Tyr) designed through 'Build Mutant' protocol of Discovery studio.

**Figure 3:**
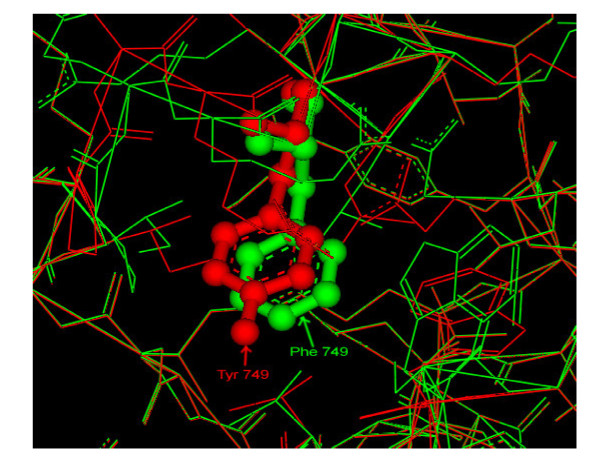
**Superimposed model of TIR domain of human TLR2 (Green) with mutated model (Red)**. Position 749 showing non-significant structural difference because of Phe749Tyr

Total grid energy of TIR model, mutated model as calculated by Delphi was 1754.014 kt and 1789.582 kt respectively. Total grid energy is the sum of the products of electrostatic potential at each grid point and the grid charges. It is calculated as the interactions of molecular charges with their own induced potential. Thus, only the differences of total grid energies for two Delphi runs are meaningful. The difference here was only 35.568 kt. Also, the difference in the coulombic energy (the energy of intramolecular electrostatic interactions in a uniform medium with the properties of the solute) was found to be 61.7 kt only. The difference in total reaction field energy (the transfer energy of a solute from a medium with the dielectric properties of the molecule to the solution) of two models was 7.98 kt only. Comparative results of Delphi calculations are summarized in Table 3. To analyze changes because of Phe749Tyr polymorphism at structure level, both the models were superimposed by using 'Align Structure' protocol on Discovery studio (figure 3). The overall RMSD calculated between two structures based on all 146 residues were 0.20 Angstrom only.

**Table 3:** Comparison of Delphi electrostatic potential calculation for TIR model and mutated model (PHE749TYR)

	**TIR model**	**Mutated model of TIR(PHE749TYR)**
Total grid energy	1754.014 kt	1789.582 kt
Corrected reaction field energy	-2123.505 kt	-2084.618 kt
		
Total reaction field energy	-24231.67 kt	-24239.65 kt
Coulombic energy	-31277.80 kt	-31339.50 kt

All calculation was performed with 33 Grid Points per Axis and Grid Filled By Solute parameter as 80%.

1 kT = 0.592 kcal/mol

## Discussion

In this study we observe that, contrary to reported associations with mycobacterial diseases in other populations [4,5,7], TLR2 polymorphisms are not responsible for the increased prevalence of TB in the Indian population. We also report for the first time the presence of a novel polymorphism in the TLR2 gene, viz. Phe749Tyr. In-silico analysis, however, showed this to be an unlikely candidate for any significant structural or functional alteration at the protein level. There is no data regarding TLR2 polymorphisms in people from the Indian subcontinent till date. Hence, our data could provide useful information on the Indian population for studies aimed at exploring the putative relevance of TLR2 SNPs not only in TB, but also in a range of other infectious and inflammatory diseases, in which the roles of TLR2 have been incriminated.

A number of previous studies have explored the occurrence of TLR2 polymorphisms in various diseased and healthy populations. The findings of these studies reveal marked variability depending on the ethnicity of the population scanned. While the Arg753Gln SNP has been reported exclusively in the Caucasian population, the Arg677Trp polymorphism has been observed in Asian and African populations. The most remarkable association with mycobacterial diseases was observed for the Arg677Trp polymorphism, which was detected in 22% of lepromatous leprosy patients in a Korean population, while being absent in patients of tuberculoid leprosy and healthy controls [5]. This polymorphism was also found to associate with TB in a Tunisian population, where the mutation was observed in 94% of 34 patients compared to 31% of 34 healthy controls [4]. Contrary to the study conducted by Kang et al [5], other authors have failed to detect these TLR2 polymorphisms in the Korean population. Examining the prevalence of TLR2 and TLR4 SNPs in bacteremic patients from Korea, Yoon et al observed no genetic polymorphism in patients or healthy controls, suggesting these SNPs to be extremely rare in this population [15]. Similarly, Ryu et al observed that TLR2 polymorphisms did not appear to be responsible for host susceptibility to lung diseases caused by non-tubercular mycobacteria in the Korean population [16]. In the Caucasian population, the Arg753Gln SNP was detected in 9.4% of the German whites, while the Arg677Trp polymorphism was not observed at all [6]. In separate studies, the Arg753Gln allele was observed among 10.34% [17] and 12.3% [18] of healthy Turkish subjects, while the Arg677Trp was not observed. Lorenz et al identified the Arg753Gln polymorphism among almost 3% of recruited subjects in another Caucasian population and speculated that the mutation could be a risk factor for developing septic shock after infection by gram-positive bacteria [19]. The absence of the TLR2 SNPs in the present study, which concurs with most of the Korean studies [15,16] and is in contrast to studies in Caucasian or African populations, could mirror the ethnic relatedness of the populations being compared.

## Conclusion

Therefore, based on our observations, it may be concluded that variations of the TLR2 genotype are not responsible for the increased susceptibility to TB in the Indian population. This calls for further studies to identify potential genetic factors, which might explain the increased burden of TB in our patients. Other population groups within the ethnically diverse Indian population also need to be recruited to understand the impact of these polymorphisms towards the development of TB in the Indian subcontinent.

## Competing interests

The authors declare that they have no competing interests.

## Authors' contributions

DB conceived of the study, participated in its designing, carried out the molecular genetic experiments, analysed the results and wrote the manuscript. SKG performed all the bioinformatics analysis on the genetic sequences obtained and participated in the drafting of the manuscript. GS was responsible for the recruitment of the study subjects and for their clinical evaluation and care. AP performed the molecular genetic experiments and assisted in data analysis. All authors have read and approved the final manuscript.
